# Supplementary material for: Oxidative Pathways of Deoxyribose and Deoxyribonate Catabolism
Source: mSystems. 2019 Feb 5;4(1):e00297-18. doi: 10.1128/mSystems.00297-18 (PMC6365646; doi:10.1128/mSystems.00297-18)
Supplement: FIG S1 [file mSystems.00297-18-sf001.pdf]

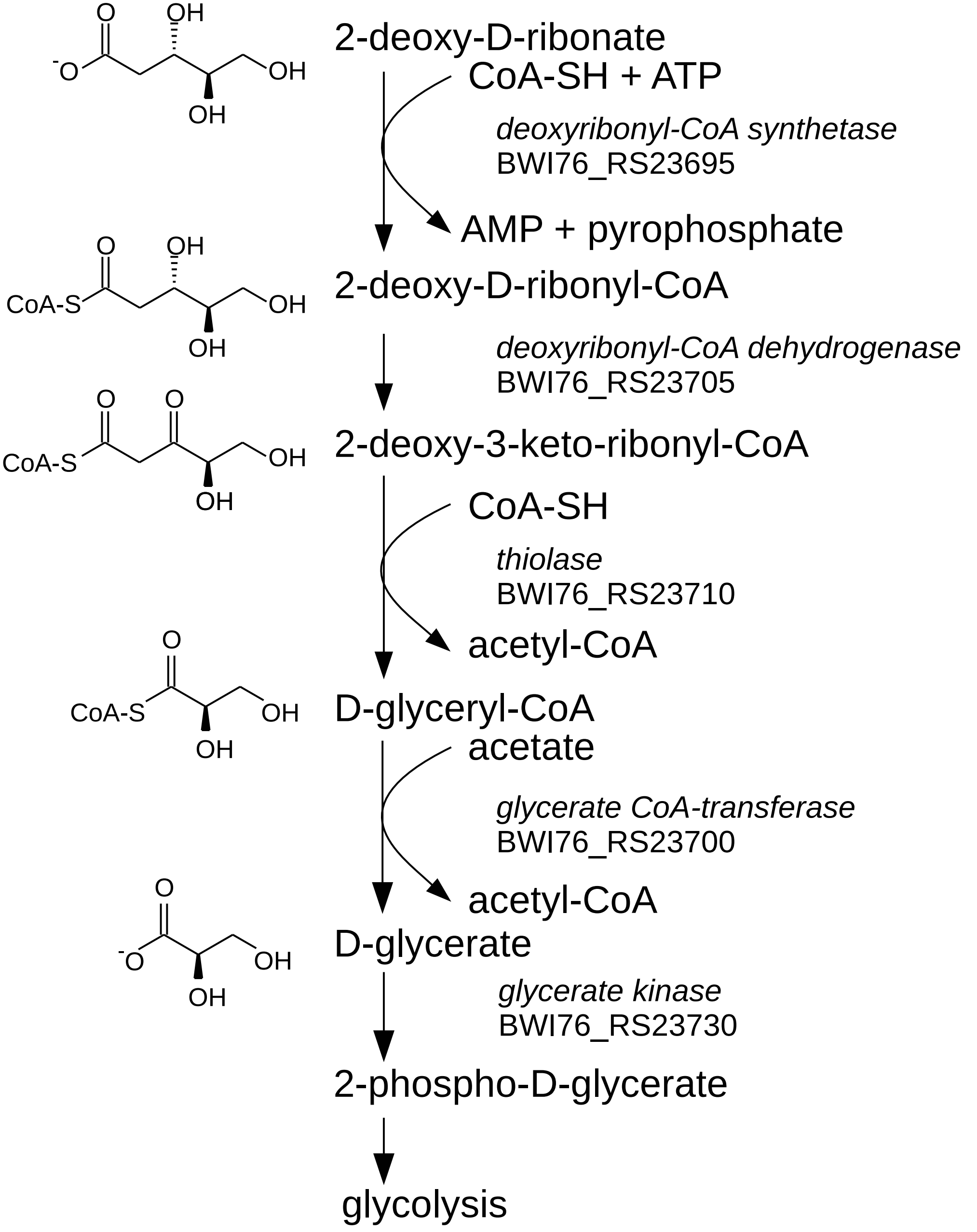

2-deoxy-D-ribonate
CoA-SH + ATP
deoxyribonyl-CoA synthetase
BWI76_RS23695
AMP + pyrophosphate
2-deoxy-D-ribonyl-CoA
deoxyribonyl-CoA dehydrogenase
BWI76_RS23705
2-deoxy-3-keto-ribonyl-CoA
CoA-SH
thiolase
BWI76_RS23710
acetyl-CoA
D-glyceryl-CoA
acetate
glycerate CoA-transferase
BWI76_RS23700
acetyl-CoA
D-glycerate
glycerate kinase
BWI76_RS23730
2-phospho-D-glycerate
glycolysis
