# Supplementary figures and images for: Oxidative Pathways of Deoxyribose and Deoxyribonate Catabolism
Source: mSystems. 2019 Feb 5;4(1):e00297-18. doi: 10.1128/mSystems.00297-18 (PMC6365646; doi:10.1128/mSystems.00297-18)

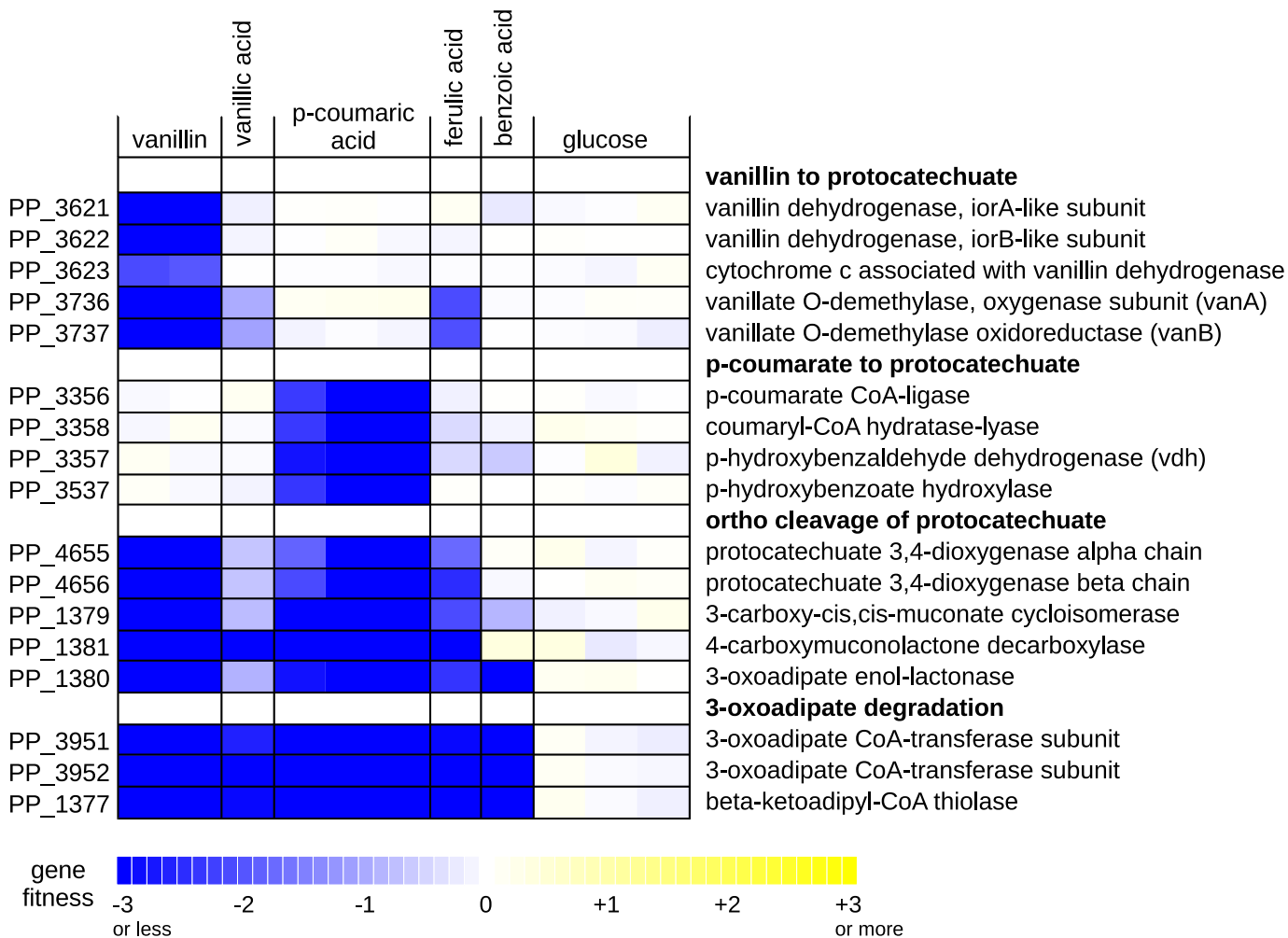

Supplement: FIG S2 [file mSystems.00297-18-sf002.pdf]

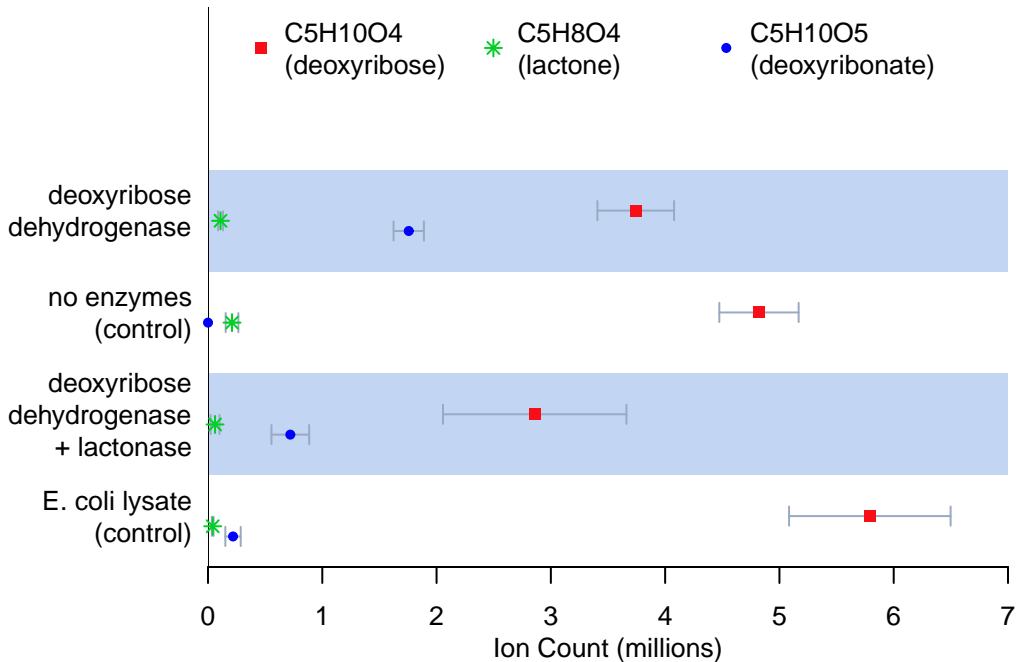

Supplement: FIG S3 [file mSystems.00297-18-sf003.pdf]

Precursor ion:  $m/z = 173.042$

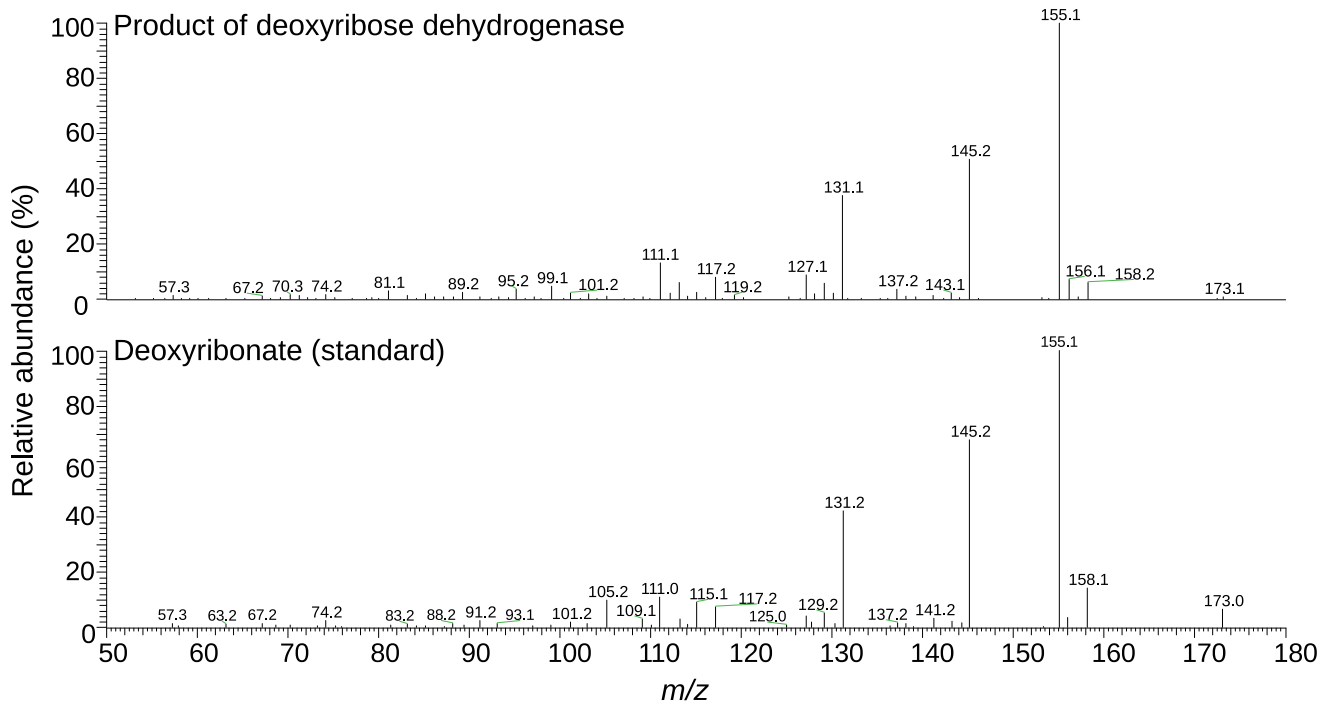

Supplement: FIG S4 [file mSystems.00297-18-sf004.pdf]

Precursor ion  $m/z = 171.02$

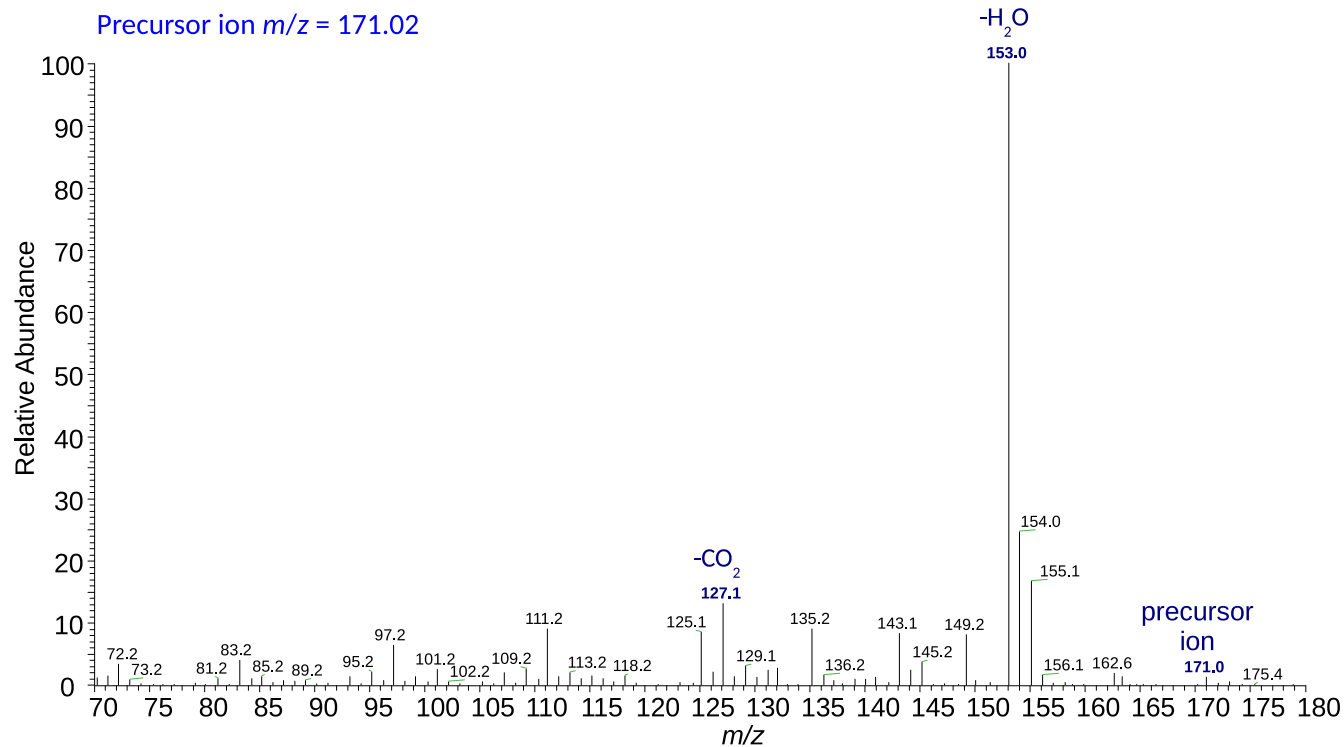

Supplement: FIG S5 [file mSystems.00297-18-sf005.pdf]
